# Cognitive Assessment in *GNAO1* Neurodevelopmental Disorder Using an Eye Tracking System

**DOI:** 10.3390/jcm10163541

**Published:** 2021-08-12

**Authors:** Federica Graziola, Giacomo Garone, Melissa Grasso, Alessandro Capuano

**Affiliations:** 1 Neurology Unit, Department of Neurosciences, Bambino Gesù Children’s Hospital, 00146 Rome, Italy; giacomo.garone@opbg.net (G.G.); melissa.grasso@opbg.net (M.G.); alessandro.capuano@opbg.net (A.C.); 2 Department of System Medicine, University of Rome Tor Vergata, 00133 Rome, Italy; 3 University Department of Pediatrics, Bambino Gesù Children’s Hospital, University of Rome Tor Vergata, 00133 Rome, Italy

**Keywords:** *GNAO1*, augmentative and alternative communication, AAC, eye tracking technology

## Abstract

*GNAO1* gene mutations are associated with a neurodevelopmental disorder characterized by developmental delay, epilepsy, and movement disorder. Eye tracking and eye movement analysis are an intriguing method to assess cognitive and language function and, to the best of our knowledge, it has never been tested in a standardized way in *GNAO1*. *GNAO1* children are usually wheelchair-bound and with numerous motor constrains, including dystonic movements and postures, heterotropia, and hypotonia, making the cognitive assessment arduous. These contribute to the burden and disability, with a high level of frustration of caregivers and patients. We have herein demonstrated that, through an eye tracking system, six *GNAO1* patients evaluated showed variable degrees of communicative intent through intentionally directed gaze. Moreover, three of these were able to complete a cognitive evaluation, and showed normal fluid intelligence and lexical comprehension. In conclusion, in *GNAO1*-related disorders, the degree of cognitive development is underestimated; eye tracking technologies may help in overcome these boundaries.

## 1. Introduction

The *GNAO1* gene, located on chromosome 16q13, encodes for an α subunit of the heterotrimeric guanine nucleotide-binding proteins (G proteins). G proteins are a large family of signal-transducing molecules composed of α, β, and γ subunits. α subunits bind the guanine nucleotides, and are capable of hydrolyzing guanosine-5-triphosphate as well as interacting with specific receptor and effector molecules. *GNAO1* is extremely abundant in brain tissue, playing an important role in brain function [1].

*GNAO1*-related disorders variably combine severe hyperkinetic movement disorders and/or early onset epilepsy [2]. Movement disorders often precipitate, causing life-threatening emergencies, and epilepsy can be drug-resistant [3]. They are usually associated with significant developmental delay, with severe language impairment and poor motor development [3]. Most patients are nonverbal and nonambulatory, and are considered to having an intellectual disability [4]. Nevertheless, the cognitive assessment of *GNAO1*-patients has never been reported in detail, most case series only reporting a descriptive assessment of the cognitive status [4]. Motor and language disability can make the cognitive assessment arduous. In fact, commonly used verbal and non-verbal intelligence tests (e.g., Wechsler scales or Raven′s Progressive Matrices) require the respondent either to provide a verbal answer or to point at it.

Augmentative and alternative communication (AAC) is a wide variety of interventions and technologies aimed at promoting communication used to compensate for an individual’s reduced communicative competence [5,6,7] Eye tracking technology (ETT) is a sensor technology used for measuring eye movements. ETT can be used as an interface for human–computer interaction, and can be applied to AAC interventions in severe neurodevelopmental and motor disorders [8]. Through ETT, subjects may control mouse pointing on a computer: the device projects a low level of infrared light, and a camera on the system captures the angle of the reflection coming back from the subjects’ eyes [7]. When the user holds their gaze on a desired location for a specific length of settle time, a mouse click is activated [9].

AAC has been widely used in infants and toddlers with disabilities in the last years [10]. Many studies have investigated particularly the use of ETT to assess cognition in individuals with severe motor apraxia [11] and developmental disorders [7]. In addition, ETT may provide benefits in the assessment of children with autism [12,13,14] or Rett syndrome [9,15,16,17,18].

In this study, we describe our experience in the assessment of the cognitive and communication profile of *GNAO1* patients by EET, as a strategy to overcome the constraints imposed by severe motor and language impairment.

## 2. Methods

### 2.1. Participants

We included all those patients with genetically proven *GNAO1* related disorders who underwent cognitive and language evaluation by ETT at our center. All procedures accorded local ethical standards.

Motor disability was classified according with the Gross Motor Function Classification System—expanded and revised (GMFCS—E&R) [19,20] and the Manual Ability Classification System (MACS) [21], respectively, for gross motor functioning and for the ability to handle objects. The Burke–Fahn–Marsden Dystonia Rating Scale (BFMDRS) was used to assess dystonia severity [22].

Efficacy of individuals’ everyday communication has been classified according to the Communication Function Classification System (CFCS) [23].

In clinical practice, patients with a CFCS level of V—meaning with a seldom effective communication, even with familiar partners—have not been addressed to ETT cognitive and language assessment. 

Adaptive skills were assessed by administering to the patients’ caregivers the Adaptive Behavior Assessment Systems (ABAS, Giunti Psychometrics, Firenze, Italy) (M:100, SD :15)—a standardized questionnaire assessing adaptive functioning in the conceptual, social, and practical domain [24]. In addition, the Pediatric Quality of Life Inventory (PedsQ, James W. Varni, Italian validation of PedsQL^TM^, Lyon, France) [25] and the Parent Stress Inventory (PSI, Giunti Psychometrics, Firenze, Italy) [26] questionnaires were used to assess patients’ quality of life and parental stress, respectively. PedsQ is expressed in percentile, and the higher the score the better the quality of life; scores of 81% or higher are considered good HRQL functioning (Health-Related Quality of Life); PSI test is also expressed in percentile, and high caregivers stress scores are above 81%.

### 2.2. Language Assessment

Lexical comprehension was assessed through a digital version of the receptive subtest of the Italian language test TFL (Phono-Vocabulary Test, Test Fono Lessicale, Edizione centro studi Erickson, Trento, Italy) [27]. The test consists in 45 tables with 4 images each: the target, a phonological distractor, a semantic distractor, and a non-related distractor. The examiner pronounces the word illustrating the target and asks the subject to point at the picture described by the word articulated. The raw score is given by the sum of the correct answers, and can be converted into percentiles for children from 2 years and 5 months of age up to 6 years of age. We converted for the maximum age possible in older children. The assessment was performed in 1 or maximum 2 visits.

### 2.3. Cognitive Assessment

A digital version of the classical Raven′s Progressive Matrices (RPM, Giunti Psychometrics, Firenze, Italy) was administered to assess fluid intelligence. Colored Progressive Matrices (CPM) were administered below, and Standard Progressive Matrices (SPM) above 11 years of age [28]. CPM contains three sections (A, Ab, and B) of 12 items each (36 total items); SPM contains five sections (A, B, C, D, and E) of 12 items each (60 total items). Each item is presented with an incomplete design and six alternative images, among which the subject must choose the one that best completes the design. The score is given by the sum of corrected answers compared with age-norm tables. The assessment was performed in 2 to 3 visits, each lasting 2–3 h with frequent breaks, allowing the children to become confident with the exercise.

### 2.4. Eye Tracking System

TFL was administrated through the digital software version of the test, and RPM tables have been scanned and adapted to the screen. Both tests were administered through a computer equipped with dual interactive screens: a vertical touch screen display (23.8 inches diagonal, 10-point touch-enabled, full HD, 1920 × 1080) positioned in line above a horizontal touch-mat display (21.3 inches diagonal, 20-point touch). The computer was connected to a Tobii Eye Tracker 4C^®^ (Tobii, Stockholm, Sweden; sampling rate 90 Hz), placed below the vertical display.

The participants comfortably sat in front of the vertical display, in their own postural system, at a distance of 50 to 95 cm from the tracker—according to manufacturer’s recommendations. No support for head stabilization was needed, as Tobii Eye Tracker 4C^®^ works in a remote mode.

**Figure 1:**
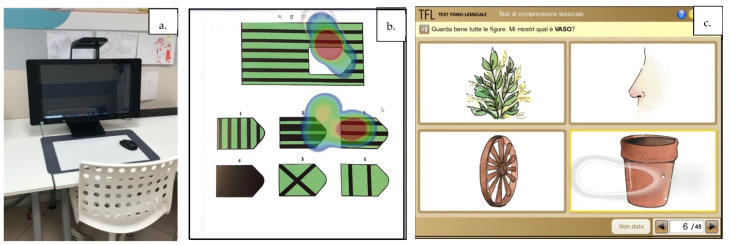
(**a**). Setting of the eye tracker system. (**b**). Scanned version of Raven′s Progressive Matrices in which the subject choses among 6 choices the best completing the design. The heatmap shows the most (red) and the least (blue) attention capturing sections. (**c**). Digital version of the Italian TFL test (Test Fono Lessicale of Italian language) analyzing the receptive Language. The subject’s eyes position is shown as a light-blue bubble on the screen. The test consists in 42 tables with 4 images each: the target, a phonological distractor, a semantic distractor, and a non-related image scrabbled in each table. The examiner shows and ask the subject to point the target with a simple question (“Guarda bene tutte le figure. Mi mostri quel’è..?, “Look carefully at all the figures. Show me where is …?”). In this example, the target is VASO (“flower vase” bottom right), the phonological distractor is NASO (“nose” top right) and the semantic distractor is PIANTA (“plant” top left).

During eye tracker use, the subject′s eyes position is shown as a light-blue bubble on the screen. For both TFL and RPM, patients were asked to fixate the image corresponding to the correct answer. Sustained fixation of a single image was recorded as a valid response, according to examiner’s evaluation; particularly, a valid response was determined according to where the participant sustained their fixation the longest. The examiner later revised the video with the recorded heatmaps in order to confirm the correct answer of the participant. The eye tracking heatmap shows the most (red) and the least (blue) attention capturing sections. See Figure 1 for further details.

## 3. Results

### 3.1. Participants

Six children with *GNAO1*-related disorders (three females, median age, range 3–15 years) underwent the ETT-based evaluation.

Five out of six patients had a GMFCS Level V, the remaining had a level IV. MACS score ranged from III (handles objects but with difficulties) to V (does not handle objects). All patients were classified with a CFCS level IV, meaning inconsistent sender and/or receiver communication with familiar partners. All patients could not perform conventional cognitive or developmental test.

All patients suffered from a mixed hyperkinetic movement disorder with prominent dystonia, and a BFMDRS median score of 33.3 (range 13–84). Patient #1 underwent bilateral globus pallidus internus deep brain stimulation surgery for refractory status dystonicus 6 months before the cognitive assessment, and patient #4, about 6 months after the assessment. Two patients were already employing a communication device, both using a voice output communication aids. None of the patients suffered from any visual or hearing conditions. See Table 1 for further clinical details.

### 3.2. Cognitive and Language Assessment

In five out of six patients, Raven’s Progressive matrices could be at least partially administered.

Three patients (patients #2, #3, and #6) completed all the items, patient #1 answered to 23 out of 60 items (40%, CPM), patient #5 completed 12 out of 36 items (33%, SPM), while patient #4 did not complete any item. Particularly, patient #1’s evaluation was interrupted due to of frequent and disturbing dystonic movements, interfering with the assessment. Patient #5’s evaluation was hampered by significant diurnal sleepiness, probably induced by a multidrug antiepileptic treatment in the context of a drug-resistant epilepsy. All three patients who completed the cognitive assessment showed a normal fluid intelligence score (above the 95° centile for their age).

Adaptive skills assessed with the ABAS test fell below two standard deviations from the mean in all patients with an extremely low level of functioning.

All six patients completed the lexical comprehension subtest of the TFL. Two patients (#1 and #4) had a score below the fifth percentile, while the scores from the other participants fell above the 10° centile, with patient #6 scoring above the 95°.

### 3.3. Quality of Life and Parental Stress

The quality of life assessed with PedsQ inventory was globally reduced in all patients compared to reference levels for age-matched controls. Parental stress index was above 80% in all patients ranging from 75 to 90%, with a mean stress index of 84%.

See Table 2 for further details.

## 4. Discussion

We have herein shown our experience in the language and cognitive assessment of a small cohort of *GNAO1* children with severe motor and communication disability. It is a pilot study propaedeutic to a deeper and longer-term prospective assessment of the cognitive profile in a larger cohort of patients with *GNAO1*-related disorders.

Our experience shows that ETT may be helpful in the assessment of patients with *GNAO1*-related disorders. All patients performed at least a part of the evaluation, showing variable degrees of communicative intent through intentionally directed gaze. The participants searched for the answers logically, with no evidence of neglect in certain areas of their visual field. Three of them proved able to perform the entire assessment, a significant result for children with severe motor and speech impairment, whose cognitive level is otherwise almost impossible to test.

Both fluid intelligence and lexical comprehension assessment produced highly variable results. Three patients showed normal fluid intelligence and lexical comprehension, suggesting that motor impairment is the major source of their poor adaptive skills. The other three patients were unable to complete the cognitive evaluation. Two of them had poor scores on lexical comprehension, while the remaining showed a receptive language in the low normal range for a preschool child.

To date, little is known about the cognitive development of patients with *GNAO1*-related disorders [4]. In most patients, lack of expressive language and poor motor development may prevent traditional cognitive assessment. The degree of cognitive development is frequently qualitatively estimated [4], possibly leading to an underestimation of cognitive level. ETT technologies can help in overcoming these challenges, at least in some patients.

In addition, a standardized cognitive assessment through ETT could be helpful to identify those patients who may take advantage from ETT-based ACC interventions in daily life. Future prospective is to assess, over a specified period of time, the use of this potentially satisfactory technology to support *GNAO1*-individuals. Similarly, to other type of diseases such as Rett Syndrome [9], quality of life and parental stress should be monitored alongside developmental improvement after proper ETT-based ACC training.

Although our results should be read in the light of the small size of our cohort and the exploratory nature of our study, we suggest that ETT-based cognitive evaluations are an interesting option for *GNAO1* patients. We showed that motor and cognitive development may be highly discordant in some *GNAO1* patients, and a normal fluid intelligence may coexist with lack of any motor milestone.

Further, longitudinal data from a larger cohort are needed to delineate the cognitive profile of children with *GNAO1*-related disorders, and to verify to what extent ETT may be useful for evaluation and rehabilitation purposes.

## Figures and Tables

**Table 1:** *GNAO1* cohort clinical and genetic characteristics, N: no, Y: yes, ICU: intensive care unit, BFMDRS: The Burke–Fahn–Marsden Dystonia Rating Scale, VOCA: Voice output communication aids, DBS: deep brain stimulation TXP: Trihexyphenidyl, y: years, CBZ: Carbamazepine, TBZ: Tetrabenazine, PB: Phenobarbital, CLD: clonidine, NPR: Niaprazine, BZPs: benzodiazepines PT: Physical therapy, SLT: Speech and language therapy, Psy: Psychotherapy, Swa: Swallowing therapy, NDT: Neurodevelopmental Disorders Therapist, and VT: Vision Therapy.

Patient	Age at Evaluation (y)	Sex	DNA Substitution	Aminoacidic Substitution	Dystonia	BFMDRS	Epilepsy	Sleep Disturbance	Status Dystonicus	PEG	DBS	Previous ACC System	Ongoing Usual Drugs	Drugs Stopped	Rehabilitation
1	15	F	c.709G > A	p.Glu237Lys	Generalized	84	N	N	Y (ICU)	N	Y (14 y)	VOCA with tablet	TXP	CBZ, TBZ, CLD, BZPs and PB	PT, SLT, PSY
2	3	F	c.709G > A	p.Glu237Lys	Generalized	16	N	N	Y (ICU)	N	N	no	TXP TBZ	no	PT, NDT, SLT, Swa
3	4	M	c.736G > A	p.Glu246Lys	Generalized	33	N	Y	N	N	N	VOCA with double communication buttons	TXP NPR	no	PT, SLT, Swa
4	8	M	c.625C > T	p.Arg209Cys	Generalized	28	focal	N	Y	N	Y (8.5 y)	no	CBZ, TBZ, CLD, BZP	Unknown	PT, VT
5	7	M	c.607G > A	p.Gly203Arg	Generalized	26	focal	Y	Y	Y (3 y)	N	no	TBZ, CBZ, Baclofen	PB, TXP	NDT and SLT
6	3	F	c.736G > A	p.Glu246Lys	Generalized	13	N	N	N	N	N	no	TBZ	Unknown	SLT, NDT

**Table 2:** *GNAO1* cohort adaptive, cognitive, and language evaluation. ABAS: Adaptive Behavior Assessment Systems, PSI: Parent Stress Inventory Test, PedsQ: Pediatric Quality of Life Inventory HRQoL Health Related Quality of Life, RPM: Raven′s Progressive Matrices, TFL: test fono lessicale (Italian language test), and na: not available.

Patient	ABAS–General Adaptive Score (M100 ds15)	ABAS–Conceptual Score (M100 ds15)	ABAS–Social Score (M100 ds15)	ABAS–Practical Score (M100 ds15)	PSI–Total Stress %	PedsQ–HRQoL %	RPM Raw Score/% or IQ	TFL–Lexical Comprehension Raw Score/%
1	45	45	51	45	75	26	23 na	33/45 <5%
2	45	45	51	45	90	42	25 >95%/130	31/45 75–90%
3	45	45	56	45	80	43	17 >95%/130	30/45 10–25%
4	45	45	55	45	90	22	na na	24/45 <5%
5	45	45	55	45	80	36	12 na	36/45 10–25%
6	45	45	56	45	90	83	30 >95%/130	42/45 >95%
